# Practice size, financial sharing and quality of care

**DOI:** 10.1186/1472-6963-13-446

**Published:** 2013-10-28

**Authors:** Rose Anne Devlin, William Hogg, Jianwei Zhong, Michael Shortt, Simone Dahrouge, Grant Russell

**Affiliations:** 1 Department of Economics, University of Ottawa, Social Sciences Building, 120 University Avenue, Ottawa, Ontario K1N 6 N5, Canada; 2 C.T. Lamont Primary Health Care Research Centre, 43 Bruyère St., Room 369Y, Ottawa, ON K1N5C8, Canada; 3 School of Primary Health Care, Monash University, 270 Ferntree Gully Rd., Bldg 1, Notting Hill VIC 3168, Australia

**Keywords:** Group size, Quality measures, Revenue sharing

## Abstract

**Background:**

Although we are observing a general move towards larger primary care practices, surprisingly little is known about the influence of key components of practice organization on primary care. We aimed to determine the relationships between practice size, and revenue sharing agreements, and quality of care.

**Methods:**

As part of a large cross sectional study, group practices were randomly selected from different primary care service delivery models in Ontario. Patient surveys and chart reviews were used to assess quality of care. Multilevel regressions controlled for patient, provider and practice characteristics.

**Results:**

Positive statistically significant associations were found between the logarithm of group size and access, comprehensiveness, and disease prevention. Negative significant associations were found between logarithm group size and continuity. No differences were found for chronic disease management and health promotion. Practices that shared revenues were found to deliver superior health promotion compared to those who did not. Interacting group size with the presence of a revenue-sharing arrangement had a negative impact on health promotion.

**Conclusions:**

Despite the limitations of our study, our findings have provided preliminary evidence of the tradeoffs inherent with increasing practice size. Larger group size is associated with better access and comprehensiveness but worse continuity of care. Revenue sharing in group practices was associated with higher health promotion compared to sharing only common costs. Further work is required to better inform policy makers and practitioners as to whether the pattern revealed in larger practices mitigates any of the previously reported benefits of continuity of primary care. We found few benefits of revenue sharing – even then the effect of revenue sharing on health promotion seemed diminished in larger practices.

## Background

After a long history of solo family practice, many Canadian family physicians (FPs) are moving towards working in groups. Approximately half of all Canadian FPs (51%) practice with other family physicians in group settings, a proportion which rises to 60% for those FPs under 35 years of age [1,2]. Increases in the prevalence of group practices have been linked to demographic shifts in the FP workforce and to government primary care reforms [2,3].

The Ontario government supports group practice models and alternative payment plans in order to address undersupply of physicians and mal-distribution of services across the province [4]. This move has also been supported by physician organizations to prevent burnout of solo physicians [5]. Studies set in the United Kingdom and the Netherlands have found higher technical quality of care but lower levels of accessibility and relational continuity in group compared to solo practices [6-8]. Many US studies have looked at the quality implications of teams, particularly in integrated primary care practices [9,10]. However, less is known about the relationship between group practice performance and the group’s internal structure – in particular the number of physicians in the practice, or how groups organize their joint finances [11-13].

In Canada, insurance for medically necessary healthcare is mandated at the national level but administered provincially. Hospital treatments and physician services are paid through public funds but physicians are essentially in private practice, leaving governments to attempt to influence the provision of care by means of incentives and disincentives. Over the past few decades there has been a push to move away from the traditional fee-for-service (FFS) practice towards other payment models which, in Ontario, include multidisciplinary community health centres (CHCs) employing salaried physicians with a focus on community needs, a capitation-based system that includes the well-established health service organizations (HSOs) and a relatively new model of physician-run group practices, the family health networks (FHNs), which incorporate extended-hour coverage, financial support for information technology and a blended remuneration formula of capitation, performance bonuses and fee for service [14].

The “Comparison of Models of Primary Care” (COMP-PC) project is a study that compared the delivery of primary care services within the four organizational models previously described in Ontario, Canada [15]. The resulting dataset (See Table 1) captured rich information on practice setting, patient and provider characteristics and organizational variables.

**Table 1:** Features of models

	***Community Health Centre (CHC)***	***Fee for Service (FFS)***	***Family Health Group (FHG)***^***a***^	***Family Health Network (FHN)***	***Health Service Organisation (HSO)***
***Year Introduced***	*1970s*	*-*	*2004*	*2001*	*1970s*
***Group Size***	*Group practice, size unspecified*	*1 Physician*	*Minimum 3*	*Minimum 3*	*Minimum 3*
***Physician remuneration***	*Salary*	*FFS*	*FFS and incentives*	*Capitation*^*b*^*with a 10% FFS compon-ent and incentives*	*Capitation*^*b*^*and incentives*
***Patient enrollment***	*Required, no roster size limit*	*Not required*	*Required, No roster size limit*	*Required, Disincentive to enroll >2,400*^*c*^	*Required, Disincentive to enroll >2,400*^*c*^
***Access***	*No specified requirements*	*No specified requirements*	*THAS Extended hours*^*e*^	*THAS Extended hours*^*d*^*Access bonus*^*e*^	*THAS Extended hours*^*d*^*Access negation*^*f*^
***Multidisciplinarity***^***g***^	*Extensive*	*None*	*None*	*Some*	*Some*
***Assistance for information technology***	*Some*	*None*	*None*	*Yes*	*None*
***Objectives/ priorities***	*Responsiveness to Population needs, multidisciplinarity, prevention, focuson underserved, community-governed*	*-*	*Accessibility*	*Accessibility, comprehensivenes, doctor-nurse collaboration, use of technology*	*Responsiveness to population needs, multidisciplinarity, health promotion, cost effective-ness*

^a^Late in 2004, the Ontario Ministry of Health (MOH) created a new model of care, the FHG, to which FFS practices could transition. A family health group (FHG) is a collaborative comprehensive primary care delivery model involving 3 or more physicians practicing together. These physicians need not be located in the same physical office space, but must be within reasonable distance of each other. FFS practices converted to this new model quickly, so that by early 2006 most FFS practices had become FHGs, and it became evident that the great majority would transition by the year end.

^b^Under capitation remuneration, family physicians received a fixed monthly fee per patient enrolled, independent of the number of visits made to the practice by that patient. The capitation fee is based on the enrolled patient sex and age. FHN physicians receive an additional 10% of the FFS structure for each visit. The latter is intended to allow better monitoring of services delivered. In 2008 all HSO were converted to family health organizations. Under that model, the practices today also receive 10% of the FFS structure for each visit.

^c^ The base capitation rate is reduced to 50% for patients enrolled to a clinician with a practice size exceeding 2,400.

^d^Each physician is required to provide at least 1, 3-hour session outside regular hours (evening/weekend) per week (up to 5 sessions per group/network/organization).

^e^An incentive bonus reduced in relation to number of visits patients make to nonspecialists outside the FHN.

^f^A penalty incurred from the capitation fee for visits patients make to nonspecialists outside the FHN. Today, HSO practices are eligible for the access bonus are not subject to negation.

^g^Multidisciplinarity refers to the presence of allied health professionals (eg, physiotherapist, social worker, and pharmacist), excluding nursing staff, but including nurse-practitioners.

We used the COMP-PC dataset to answer two research questions: 1) what is the relationship between practice size and the quality of primary care? and 2) what is the relationship between revenue sharing agreements and the quality of care delivered by group practices? We also address the combined effect of group size and revenue sharing on quality of care.

### Theoretical framework

Both group size and sharing can influence performance. Once group practices are formed, whether and what revenues are shared can provide different incentives affecting the quality of care. Existing theoretical literature is unclear as to the final effect of revenue-sharing on the quality of the health services provided by a practice. Classical economic theory suggests that revenue-sharing leads to a decline in the quality of care because physicians in a group may free-ride on the efforts of their colleagues [16-20]. This phenomenon is more likely to occur when patient demand is at least partially a function of the average quality of care provided by the group. However, others have shown that sharing revenues may facilitate quality control and hence enhance the performance of medical group practices [21]. It may also foster closer links between individual physician and group income hence encouraging mutual help activities among physicians that could promote quality [22].

The size of a group practice may influence output performance if there are economies of scale or scope that can be realized by larger groups [18,21]. Empirically, the link between group size and quality of patient care has not been firmly established [13,23]. Some have discussed what the “ideal size” for a practice might be, but this question remains unresolved [7,24-27].

Finally, it is possible that the size of a group may interact with the sharing arrangement to influence performance. For instance, the free-riding tendencies emanating from a revenue-sharing arrangement may be enhanced with the size of the group.

## Methods

### Design

The COMP-PC project was a cross-sectional, mixed-methods study set in primary care practices in Ontario, Canada, between October 2005 and June 2006. Data collection methods have been described elsewhere [15] and are summarized below. The study was approved by the Ottawa Hospital Research Ethics Board (2010467-01H) and the Bruyère Research Institute Research Ethics Board (M16-12-020).

### Sample

The COMP-PC project aimed to recruit 35 practices of each service delivery model from a sampling frame that included all known Family Health Networks (94 FHN), Community Health Centers (51 CHC) and Health Service Organizations (65 HSO) in Ontario. The fee-for-service (FFS) sampling base of 155 represented a random sample extracted from a list of 1,884 practices. Practices were excluded if they did not offer primary care services for adults, had belonged to their current service delivery model for less than one year or if fewer than 50% of the sites’ providers consented to participate in the study [15]. There are 137 practices in the COMP-PC dataset.

This study uses data from the group practices identified in the COMP-PC dataset. Group practices were defined as those having more than one Full-Time-Equivalent (FTE) family physician and which shared at least four of the following five organizational elements: office space, staff, expenses, patient records or on-call duties. The results below were calculated using 84 group practices: 32 CHCs, 21 FFSs, 20 FHNs and 11 HSOs. Group size refers to the number of FTE physicians in the practice.

Data were gathered by patient, provider and practice surveys and chart audits. Patients were eligible if they were at least 18 years old, were patients of a consenting provider, were cognitively competent, and not acutely ill. Sampling aimed at recruiting between 30 and 50 participants at practice site waiting rooms. Patients completed most of the survey in the waiting room prior to their visit. Health promotion activities were measured immediately after their visit. Of the 5,361 patients recruited, 3,392 attended the 84 group practices.

Chart reviews were performed on randomly-selected records of participating providers. Charts represented patients aged 17 years or older, who attended the practice for more than two years, and who visited the practice in the year prior to the chart review. Thirty charts at each practice site were sampled for a total of 2,520 individuals in the 84 group practices.

### Instruments

Patient, provider, and practice surveys were adapted from the abridged adult version of the Primary Care Assessment Tool (PCAT) [28]. Information on revenue sharing was collected in the practice survey. A chart abstraction tool gathered information on prevention and chronic disease management quality.

### Outcome measures

Our analysis was oriented to a framework [29] that conceptualized primary care performance as having two components: health care service delivery (measured by scales for accessibility, comprehensiveness, and continuity) and technical quality of care (measured by scales for health promotion, disease prevention and chronic disease management (CDM)). These concepts were captured using different tools. The healthcare service delivery scales used were derived from those in the Primary Care Assessment Tool (PCAT), an instrument developed to measure the quality of primary care services. The full version of the PCAT was validated in 2001 [28]. The technical quality of care scales for disease prevention and CDM were based on chart audits for recommended manoeuvres, while the seven item question addressing health promotion activities was adapted from the PCAT and is consistent with the recommendations of the Canadian Task Force on Preventive Health Care [30,31]. Table 2 describes the data sources, item pools and scoring methods used for the scales.

**Table 2:** Health outcomes scales

**Scale**	**Data source**	**Item pool**	**Scoring**
Accessibility	Patient survey	Scale of 4 Likert items covering patient-perceived first-contact accessibility.	Score/Total Possible Score
Comprehensiveness	Practice survey	List of 15 important clinical services drawn from procedural, counseling, women’s health and diagnostic services.	# Services offered/15
Continuity	Patient survey	Scale of 4 Likert items covering patient-provider knowledge, relational continuity and ability to be seen by most knowledgeable provider.	Score/Total Possible Score
Chronic Disease Management	Chart review	Comparison of tests and services received against Canadian clinical practice guidelines for patients with congestive heart failure, coronary artery disease and/or diabetes.	# Procedures Received/ # Eligible Procedures (11 assessed)
Disease Prevention	Chart review	Adherence to the Canadian Task Force on Preventive Health Care (CTFPHC) guidelines for preventive maneuvers.	# Procedures Received/ # Eligible Procedures (6 assessed)
Health Promotion	Patient survey	Adherence to the CTFPHC guidelines for health promotion.	# Areas/7

### Data analysis

An Ordinary Least Squares procedure using SPSS-PC version 16.0 was applied to the following equation:

$$Q_{i}{=\beta }_{0}{+\beta }_{1}{Z+\beta }_{2}{D+\beta }_{3}{S+\beta }_{4}{D*S+\beta }_{5}M_{j}+\epsilon$$

where: Q_i_ (i = 1…6) represents the six different measures of quality of care; β_k_ (k = 0…5) are estimated coefficients; Z is a vector of patient, physician and practice characteristics; D is a dichotomous variable which takes on the value of 1 if the practice engages in revenue sharing and 0 otherwise; S represents the size of the group, while M_j_ (j = 1…4) represents the four practice models (FFS, CHC, FHN and HSO).

We are particularly interested in the signs of β_2_ (the impact of revenue sharing), β_3_ (the impact of group size) and β_4_ (the interaction of revenue sharing and group size).

Two different specifications were employed. The first includes all four practice models. The second specification excludes CHCs because their physicians are salaried and do not have a revenue-sharing arrangement, leaving a sample of 52 practices. Therefore, the first set of regression results focuses on the impact of practice size, defined here as the logarithm of the number of full-time-equivalent family physicians [32], on our six quality indicators, while the second focuses on revenue-sharing and practice size. Only the patient, physician and practice characteristics that were significantly associated (at an alpha level of 0.10) with quality of care were kept in the regression. The logarithm of size, as well as practice model indicator variables, was included in all regressions.

The equations with comprehensiveness as the dependent variable have the practice as the unit of observation (84 and 52 observations for the practice size and revenue sharing analysis, respectively). The other five quality measures use the patient as the unit of observation and hence have a much larger sample size (3,392/2,270 for Access, Continuity and Health Promotion and 2,520/1,680 for Chronic Disease Management and Disease Prevention, for the two analyses respectively).

## Results

Table 3 reports the means and standard deviations across model type for each variable used in the analysis. Several differences clearly exist in the patient profiles, practice characteristics and physician profiles. For instance, 73% of CHC patients were female while only 51% of HSO patients were; electronic medical records were present in 2 of 21 FFS practices whereas 7 of 32 CHCs used this technology; and more female physicians are found in CHCs as compared to all other models.

**Table 3:** Descriptive statistics

	**CHC**	**FFS**	**FHN**	**HSO**
**Patient profile n**	1122	816	1015	439
Age (years)	46(17)	49 (17)	51 (16)	51 (17)
% Sex female	73 (45)	71 (45)	67 (47)	59 (49)
% Insured in Ontario	92 (28)	96 (20)	97 (18)	97 (18)
% Ethnicity white	84 (37)	91 (29)	96 (19)	96 (19)
% Born in Canada	70 (46)	74 (44)	86 (35)	83 (37)
Duration in Canada (immigrants only)	19 (17)	32 (17)	38 (17)	37 (15)
% Household with income greater than LICO	73 (45)	90 (30)	91 (29)	91 (29)
% High school graduate	73 (44)	83 (38)	81 (39)	81 (39)
% whose main provider is a Nurse Practitioner	21 (41)	0 (5)	0 (0)	2 (15)
**Practice characteristics**				
n	32	21	20	11
EMR exists	7	3	10	2
Patients per physician and nurse practitioner	764 (472)	1830 (1252)	1282 (804)	1527 (811)
Booking interval for routine visit (minutes)	24.69 (4.24)	13 (1.99)	14.02 (2.53)	13.18 (3.54)
# Family Physicians (FTE)	3.41 (.85)	3.43 (1.75)	4.41 (1.75)	2.95 (1.42)
# Nurse Practitioners (FTE)	2.89 (1.94)	0.72 (1.11)	2.51 (2.12)	1.41 (1.24)
% existing Allied Health Professionals	28	3	3	6
% with Nearest hospital > 10 km	7	2	2	2
Rurality index	11.33 (15.11)	9.91 (20.05)	21.80 (25.79)	9.12 (11.01)
Practice shares revenues	-	1	4	8
**Family physician profile n**	166	42	44	42
Years since graduation (as of 2006)	19.96 (6.6)	20.61 (7.15)	23.53 (6.61)	28.61 (9.94)
% Female providers	57.4 (35.4)	44.7 (44.6)	35 (40.6)	20 (37.6)
% Provider foreign trained	8 (13)	12 (27)	2 (10)	12 (30)
% Provider with CFPC degree	78.88 (45)	67 (47)	65 (48)	60 (49)

*LICO*, Low income cutoff; *EMR*, Electronic medical records.

Each column reports the mean value, with standard deviation in the parentheses.

Table 4 reports the regression results for the equations evaluating group size for each of our six quality of care indictors. Positive associations were found between the logarithm of group size for access [1.17% (−0.04, 2.3), P = 0.043], comprehensiveness [9.7% (3.5, 15.8), P = 0.002], and disease prevention [3.3% (−0.5, 7.1), P = 0.093]. Group size was negatively associated with continuity [−2.2% (−3.2, -1.3), P = 0.01]. There were no significant associations between practice size and CDM or health promotion.

**Table 4:** Regression results of group size and quality of care

**Dependant variable**	**Access**	**Comp-ness**	**Continuity**	**CDM**	**Disease prevention**	**Health promotion**
Logarithm (FP)	0.01169^**^	0.097^***^	−0.02246^***^	−0.0198	0.03282^*^	0.00793
Patient = female	0.01868^***^		0.012622^**^	−0.07747^**^	0.1886^***^	−0.02027^**^
Patient Age	0.00089^***^		0.001019^***^	0.00326^***^	−0.00392^***^	−0.00107^***^
Chronic disease			0.03396^***^		−0.04204^*^	0.02832^***^
Selfhealth > fair	0.0302^***^					
% of Female FPs					0.08214^***^	0.06529^***^
% of Canadian educated FPs	0.0708^***^		0.025236^*^		−0.04379^*^	
Years since grad (FPs)	0.0015^***^		0.000883^**^			−0.001955^***^
Rurality Index	−0.00073^***^		−0.00024^*^		−0.00142^***^	
# NP			−0.00814^***^		−0.04123^***^	
Other HP Exist		0.014^***^				
other Hospital >10 km	0.0518^***^	0.115^***^	−0.013502^**^			
EMR					0.034216^*^	
**Model (CHC ref)**						
FFS	−0.0219^***^	0.036	−0.01108	−0.13955^***^	−0.16369^***^	−0.01945^*^
FHN	0.000344	0.03	−0.01636	−0.14324^***^	−0.05882	0.003011
HSO	0.0614^***^	0.009	0.012943	−0.10884^**^	−0.20511^***^	−0.003897
Constant	0.5659^***^	0.476^***^	0.783532^***^	0.58627^***^	0.80894^***^	0.26988^***^
R^2^		0.634				
# cases	3392	84	3392	2640	2640	3392

*Comp-ness*, Comprehensiveness; *NP*, Nurse practitioner; ^*^indicates significance at the p < 0.10 level, ^**^indicates significance at the p < 0.05 level, ^***^indicates significance at the p < 0.01 level.

Table 5 shows the results of the second regression equation which includes a variable indicating whether the physicians in the practice have a revenue-sharing arrangement. Practices that share revenues were found to deliver superior performance for health promotion [9.63% (2.5,16.7), P = 0.008] compared to those not sharing revenue. The presence of a revenue-sharing arrangement was not significantly associated with the other five measures of quality.

**Table 5:** Regression results of revenue sharing and quality of care

**Dependant variable**	**Access**	**Comp-ness**	**Continuity**	**CDM**	**Disease prevention**	**Health promotion**
Share revenue	0.019856	0.16	−0.009157	0.159041	0.021494	0.0963^***^
LnFP	0.00419	0.09^**^	−0.029084^***^	0.098929	0.052783	0.02344^**^
Share *LnFP	−0.008815	−0.07	−0.001901	−0.12157	−0.03369	−0.05371^**^
Patient = female	0.020599^**^		0.014561^***^	−0.069235^*^	0.198467^***^	−0.03505^***^
Patient Age	0.001236^**^		0.000924^***^	0.003226^**^	−0.00363^***^	−0.00059^*^
Chronic disease			0.033177^***^			
% of Female FPs					0.149436^***^	0.07312^***^
% of Canadian educated FPs	0.079598^***^		0.060374^***^	0.300983^***^		
Years since grad (FPs)	0.001713^***^		0.00296^***^			−0.00309^***^
Rurality Index	−0.000572^***^					
# NP			−0.05069^***^		−0.0852^**^	
Other HP Exists		0.172^***^				
Other Hospital >10 km	0.047203^***^	0.113^*^				
EMR			0.029992^***^			
**Model (FFS ref)**						
FHN	0.035123^***^	−0.009	−0.001075	−0.05553	0.10145^***^	0.01585
HSO	0.076212^***^	−0.1	0.023328^*^	0.023554	−0.02632	
Constant	0.546303^***^	0.512^***^	0.696944^***^	0.030827	0.51964^***^	0.25253^***^
R^2^		0.713				
# Cases	2270	52	2270	1680	1680	2270

*NP*, Nurse practitioner; ^*^indicates significance at the p < 0.10 level, ^**^indicates significance at the p < 0.05 level, ^***^indicates significance at the p < 0.01 level.

There was a significant negative effect on health promotion from interacting the presence of a revenue-sharing arrangement with the number of FTE physicians (in logarithms). The negative sign for the interaction term across all other quality measures may be interpreted as suggesting that revenue sharing has a smaller (or more negative) effect on quality when undertaken by larger groups: in other words, the presence of more physicians in the group may lead to a dampening of any positive effects from revenue-sharing per se.

Once again, the size of the practice matters for some quality indicators: Comprehensiveness, Continuity and Health Promotion. Size also mattered for the first two of these indicators in the larger specification which included CHCs.

## Discussion

Our analysis provides critical insights into the relationship between the number of family physicians in group practices and revenue sharing arrangements on six measures of quality primary care.

### Diminishing returns and group size

The estimated associations between the logarithm of group size and our six quality of care measures means that an increase in the number of full-time equivalent physicians from two to four (representing a one unit increase in FTEs on the logarithmic scale) would, on average, be associated with greater comprehensiveness – providing about 1.5 more services out of the 15 captured by this variable; a small (1.2%) improvement in patient reported access, and a 3.3% improvement in the preventive care scale (so that, for those patients eligible for the six manoeuvres, one additional manoeuvre would be performed for every five patients). In addition we found that our measure of continuity would fall by 2.3% with this same increase in FTE physicians, suggesting that patients would perceive a diminished depth in their relationship with their own provider.

If yet another two FTE physicians were to join the group (i.e., the number of physicians increases from four to six), then the comprehensiveness score, access score and disease prevention score would still all increase but by smaller increments (equivalent to 0.59 services and 0.6% of the access scale and 1.65% of the disease prevention scale), and the continuity score would fall, again by a smaller increment (0.8% of the continuity scale). These findings suggest a concave relationship between group size, comprehensiveness, access and disease prevention, meaning that gains in these measures of quality of care become increasingly difficult to achieve as the number of FTE physicians increase. Similarly, the attendant loss of continuity associated with more physicians becomes less and less pronounced.

### Revenue sharing and its interaction with group size

Cross tabulations on the sample show that as group size increases, the practice is more likely to be involved in a revenue sharing arrangement, and hence an “individual physician is less likely to have to bear the financial consequences of his decision” [20]. FHNs and HSOs are more likely than FFS practices to choose revenue sharing. Our regression results support the idea that revenue sharing encourages “mutual help” activities (such as offering advice, encouragement, or resources to fellow physicians) that increase group productivity but do not directly generate revenue for individual physicians [22]. Revenue sharing, therefore, may lead physicians to be less focused on the volume of patient visits, leading to higher quality patient interactions. As pointed out by Campbell and colleagues [7], team climate reflects how physicians actually work together and how much support is given towards maintaining high standards of care. Our work suggests that observing that practices share revenue might be a good proxy for the existence of effective teamwork and hence for the presence of high quality of care.

However, our results also suggest that bigger is not always better. As practice size increases, the incentive to improve the quality care is weakened, possibly due to the diminishing return from adjusting the group scale or due to an increased inclination to free ride on other members of the bigger group. Furthermore, when we compare the magnitude of the independent effect of revenue sharing with the interaction effect with group size, the former always dominates the latter, meaning that revenue sharing may counteract some of the barriers to high quality care that Hulscher and colleagues describe for large groups [33].

### Group size, access and continuity

Haggerty et al. [27] studied primary care group practices in Québec and found that clinics with 10 or more physicians suffered declines in accessibility and continuity. This contrasts with our results, which found an immediate decline in continuity with larger group sizes, and an increase in accessibility rather than a decrease. Their finding supports a policy of encouraging primary care groups of between six and eight physicians, while our study reveals that not all measure of care improve with group size and hence no clear optimal group size emerges. Of course, discussions of optimal group size must go hand in hand with information on costs of care, which is outside of the objective of this paper.

### Group size, health promotion and disease prevention

The positive association between group size and health promotion may arise for a number of reasons, not the least of which is that larger groups are more likely to have nurses and nurse practitioners who are often involved in health promotion [34]. In a similar vein, our finding that disease prevention scores also increase with group size is in line with Pham et al. who report that larger primary care groups provide better preventive care [33,35].

### Chronic disease management

We found that as group size increases, practices tend towards higher CDM scores, but this effect was not statistically significant. Elsewhere our group has published an investigation of chronic disease care using COMP-PC data, and we found that high quality CDM was associated with practices of four or fewer physicians [36]. Given the significantly different sample sizes (n = 132 in the CDM analysis paper, compared to n = 77 in the current analysis) it is difficult to compare these results directly. It does, however, raise the important question of how group size should be measured. Additional econometric work in this area would be helpful.

### Limitations

Working with data always comes with limitations. The first that needs to be mentioned concerns how to measure practice size. In this paper we used FTE physicians, however outcome measures like comprehensiveness might be responsive to the actual number of physicians, regardless of work load. Our results were invariant to different definitions and transformations of FTE group size, suggesting that our findings with respect to the impact of group size on quality of care are relatively insensitive to measurement specifications.

A second limitation is that we did not have detailed information on the exact nature of the revenue sharing that occurred within practices. Hence we could not be certain whether billings were divided equally, by seniority, or by another method entirely. More detailed information on revenue-sharing schemes would allow a better study of the relationship between physician-level incentives and quality of care.

We are also limited by the available information on other aspects of the internal functioning of groups, like physicians’ ability to learn and adapt to group dynamics and synchronize practice redesign [37]. The size of the group may not reflect how well the group network functions, which further limits our analysis [13].

Finally, this is a cross sectional study so we can only report associations but cannot infer causality. It is possible that physicians who choose to practice in certain settings do so as a result of unmeasured factors (such as personality type or practice style) that could also influence clinical performance.

## Conclusions

Although we are observing a general move towards larger primary care practices, surprisingly little is known about the influence of size on quality of care. Despite the limitations of our study, our findings provide some preliminary evidence of the tradeoffs inherent with increasing practice size. Further work would be necessary in order to better inform policy makers and practitioners as to whether the pattern revealed in larger practices mitigates any of the previously reported benefits of continuity of primary care [38].

We were able to undertake the analysis in this paper because of access to a unique data set. Further work, however, is definitely warranted. Larger practice samples (as may be found in health administrative databases) would be required to tease out the complex relationships between practice organizational structures, including how physicians share finances, and quality and quantity of services provided. Understanding the link between practice size and quality of services is clearly essential in order to evaluate the effectiveness of policies designed to encourage group practices and to provide guidance for appropriate and better health care decisions.

## Abbreviations

FP: Family physician; FFS: Fee-for-service; CHC: Community health centre; HSO: Health services organization; FHN: Family health network; COMP-PC: Comparison of models project; FTE: Full time equivalent; PCAT: Primary care assessment tool; CDM: Chronic disease management.

## Competing interests

The authors declare that they have no competing interest.

## Authors’ contributions

RAD led the analysis and writing of the paper. WH conceived of and led the original Comparison of Models project and participated in the interpretation of the results and writing of the paper, JZ performed most of the analyses and participated in the interpretation of the results and writing of the paper, SD led the data collection for the original Comparison of Models project and participated in the interpretation of the results and writing of the paper, GR assisted with the data collection in the original project and participated in the interpretation of the results and writing of the paper and MS assisted with the analysis and writing of the paper. All authors read and approved the final manuscript.

## Pre-publication history

The pre-publication history for this paper can be accessed here:

http://www.biomedcentral.com/1472-6963/13/446/prepub
